# Low vision care for patients with glaucoma: there is more you can do!

**Published:** 2022-01-31

**Authors:** Karin Van Dijk, Aimbora Kristonsia Kimaro, Heiko Philippin

**Affiliations:** 1 Low Vision Advisor – Global Level: CBM, Deventer, The Netherlands.; 2 Optometrist and Low Vision Therapist: Kilimanjaro Christian Medical Centre Eye Department, Moshi, Tanzania.; 3 Clinical Research Fellow: International Centre for Eye Health, London School of Hygiene & Tropical Medicine, UK. Global Advisor for Inclusive Eye Health/Research & Training: CBM, Bensheim, Germany and Glaucoma Specialist: Eye Center, Medical Center, University of Freiburg, Germany.

**Some patients with glaucoma will experience permanent vision loss. The correct low vision advice and support will enable them to carry out their daily activities with greater ease and comfort.**

**Figure:**
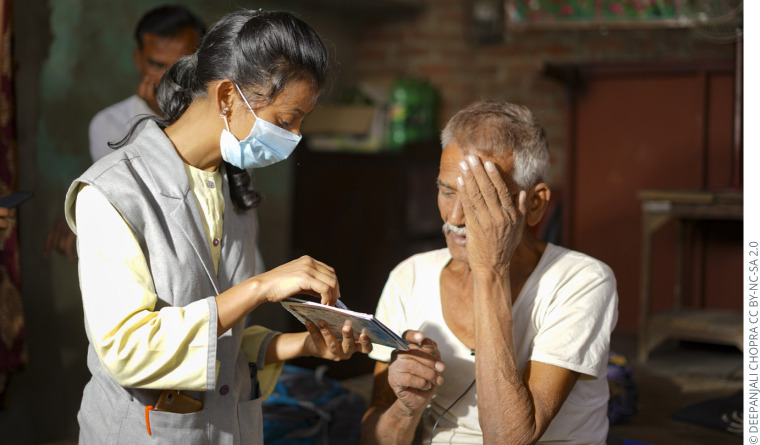
Assess for and correct presbyopia in older glaucoma patients before starting other low vision interventions. **INDIA**

People with glaucoma have specific vision-related problems that will affect their daily activities in various ways (Table 1).^1^ Learning about patients’ needs and challenges is the first step in offering them useful advice on suitable interventions.

**Table 1:** The vision-related challenges of people with glaucomatous vision loss with examples of how daily activities may be affected.^1^

Vision-related problems	Examples of daily activities affected
Loss of peripheral (side) vision	Detecting people or objects only when they appear in the central visual field; bumping or stumbling into objects
Paracentral scotomas or blurred parts	Reading is often slow and words may be missed
Reduced ability to adapt from light to dark and dark to light	Moving around during sudden changes in lighting is more difficult
Trouble seeing in the dark	Orientation at dusk or at night is limited
Reduced (distance) visual acuity	Unable to recognise people in the distance (but may still see small details)
Light sensitivity (especially to glare)	Working outside when the sun shines is difficult; cannot see well inside with glare from window light
Impaired contrast sensitivity	Unsteady navigating on uneven terrain; tripping on steps; limited interpretation of facial expressions

First ask questions (and observe) what your clients need and what they now find difficult when performing their daily activities. Use different tests (clinical and functional) to assess distance and near visual acuity, visual field, contrast sensitivity, and light sensitivity. The CEHJ article ‘When someone has low vision’ lists useful methods.^2^ Check what support there is at home, at work, at school and in the community. This will help you to advise them on training and interventions. For example, if someone needs to be guided at night when walking to the local shop, it would be helpful to train the client and a family member or friend in a safe way of guiding.^3^ Remember to correct presbyopia in older patients before starting other interventions.

## What interventions can help?

Here are some ideas you can suggest to your client.

- Sunglasses can help to reduce the effects of glare and improve contrast. Try a few different coloured lenses to find the ones that work best (see Case study 1, opposite).
- Improve lighting. Consider quantity, type, and direction. E.g., try a reading lamp with a flexible arm in a position that avoids creating glare.
- Ensure enough ambient lighting in dimly lit rooms and prevent large differences in lighting levels.
- Reduce glare by closing curtains or changing position so that you have less excess light.
- Add contrasting strips to steps. Line the borders of the garden with bricks painted white.
- Remove clutter and dispose of little-used items in your kitchen.
- Always carry a torch with you.
- Move closer to the TV.
- Use felt-tipped pens, which are bolder and easier to see.
- Enlarge the labels on your medication or colour code them.

Provide (or advise the client to undergo) compensatory visual field training to enable people with visual field defects to improve navigation and avoid obstacles. For example: “Pause regularly when walking and move your head slowly up and down, then from left to right, to scan the area in front of you. For example, if you scan the area before crossing the street, you may notice a car parked on the street corner which you did not see when looking straight ahead. Then you can avoid bumping into it.”

Refer the client to appropriate peer support groups and to counselling services; these can be of real benefit to them.

Other interventions that some (but not all) people need:

- Orientation and mobility training to learn to walk using a white cane, for people who have lost all their vision or have very low visual acuity.
- Magnifiers, after refraction, correction of presbyopia and prescription of glasses has been done (see Case study 2). These can include **optical magnifiers** (often only low to medium magnification is possible due to the limited visual field) and **smartphone apps** that magnify.^4^ A **handheld video magnifier** can offer a significantly larger field of view at a given level of magnification and contrast can be enhanced.
- Text-to-speech software, e.g., the free application ‘Non-visual Desktop Access’ (NVDA).^5^
- Reverse telescopes: objects look smaller so that more information fits into a small field of vision (only people with a good distance acuity will benefit).

**Case study 1**

The client is a 68-year-old woman with glaucoma in both eyes. She has no spectacles and reports problems moving around at night and when the sun shines. She also finds it difficult to see what she is doing when gardening.

**Main need identified**

Better vision to move around and do her gardening.

**Assessment**

- She has no spectacles.
- Uncorrected distance visual acuity (VA): 6/60. With a -6.00D correction, VA is 6/36.
- Uncorrected near VA: 2M at 9 cm; best corrected near VA: 1.25M at 10 cm (near add +3.00D).
- Contrast sensitivity: 10%.
- Assessed for magnifier but she does not need it, she says.

**Interventions**

- Bifocals and cover spectacles (dark glasses that fit over her distance glasses; this reduces glare and too much light)
- Ensure even lighting levels throughout the house, to reduce risk of falling over (unexpected) obstacles.

**Client’s comment**

“Now I can do my gardening again, and I can do it in comfort.”

**Figure:**
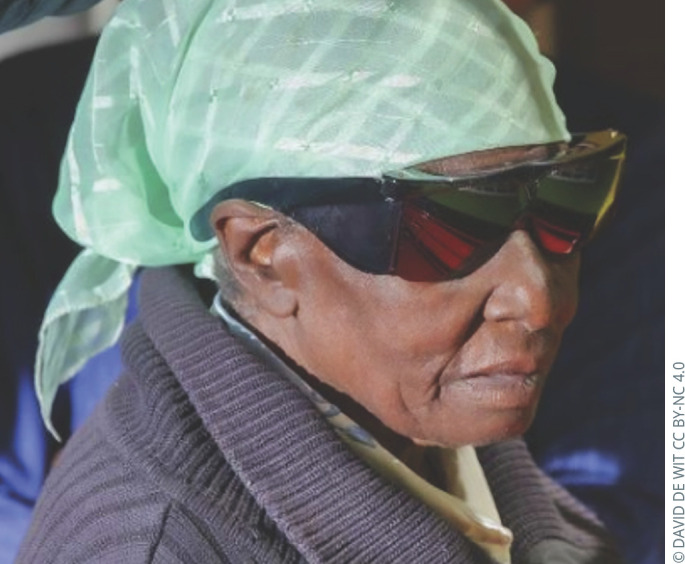
Woman wearing dark cover spectacles. She can now work comfortably in her garden. **ZIMBABWE**

**Case study 2**

The client is a 15-year-old boy with pseudophakic glaucoma in both eyes. He attends primary school and has been on timolol eye drops since infancy.

**Assessment**

He only has vision in his left eye: uncorrected 6/36 and with spectacles 6/18.

**Interventions**

Optical magnification (used since 2014):

- Monocular 4x telescope which gives 6/6 distance VA.
- 20.00D (6x) handheld magnifier which gives near VA of 0.8M at 25 cm.

**Client’s comments**

“Before 2014, even though I had glasses, I still had difficulties in class, e.g., in reading the blackboard and small print in textbooks. Since I was given the telescope and the magnifier, I can read the blackboard without asking for help and all small print.

“These devices make me more confident and my position in class has increased from 65th out of 105 to 14th out of 101 in a school with normally sighted peers.”

**Figure:**
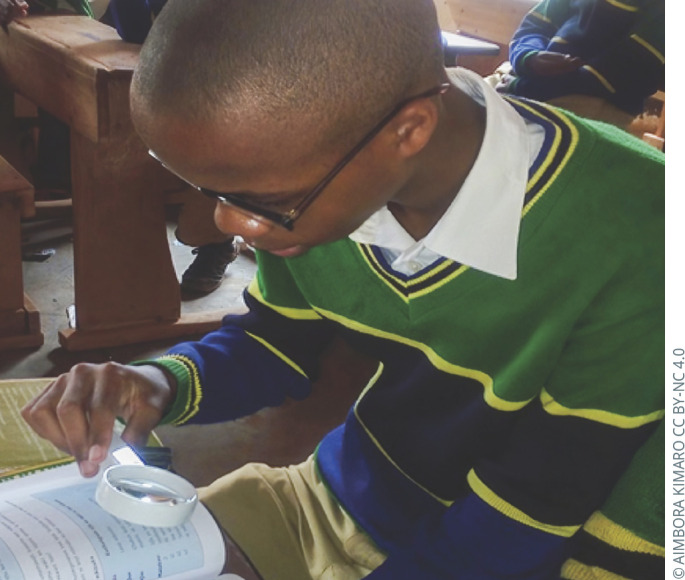
15-year-old reading a schoolbook with 20D hand magnifier. **TANZANIA**
